# The Problematic Concept of Native Speaker in Psycholinguistics: Replacing Vague and Harmful Terminology With Inclusive and Accurate Measures

**DOI:** 10.3389/fpsyg.2021.715843

**Published:** 2021-09-30

**Authors:** Lauretta S. P. Cheng, Danielle Burgess, Natasha Vernooij, Cecilia Solís-Barroso, Ashley McDermott, Savithry Namboodiripad

**Affiliations:** ^1^ Department of Linguistics, University of Michigan, Ann Arbor, MI, United States; ^2^ Department of Psychology, University of Michigan, Ann Arbor, MI, United States; ^3^ Department of Anthropology, University of Michigan, Ann Arbor, MI, United States

**Keywords:** research methods, native speaker, psycholinguistics, language experience, multilingualism

## Abstract

Though the term NATIVE SPEAKER/SIGNER is frequently used in language research, it is inconsistently conceptualized. Factors, such as age, order, and context of acquisition, in addition to social/cultural identity, are often differentially conflated. While the ambiguity and harmful consequences of the term NATIVE SPEAKER have been problematized across disciplines, much of this literature attempts to repurpose the term in order to include and/or exclude certain populations. This paper problematizes NATIVE SPEAKER within psycholinguistics, arguing that the term is both unhelpful to rigorous theory construction and harmful to marginalized populations by reproducing normative assumptions about behavior, experience, and identity. We propose that language researchers avoid NATIVE SPEAKER altogether, and we suggest alternate ways of characterizing language experience/use. The vagueness of NATIVE SPEAKER can create problems in research design (e.g., through systematically excluding certain populations), recruitment (as participants’ definitions might diverge from researchers’), and analysis (by distilling continuous factors into under-specified binary categories). This can result in barriers to cross-study comparison, which is particularly concerning for theory construction and replicability. From a research ethics perspective, it matters how participants are characterized and included: Excluding participants based on binary/essentialist conceptualizations of nativeness upholds deficit perspectives toward multilingualism and non-hegemonic modes of language acquisition. Finally, by implicitly assuming the existence of a critical period, NATIVE SPEAKER brings with it theoretical baggage which not all researchers may want to carry. Given the issues above and how ‘nativeness’ is racialized (particularly in European and North American contexts), we ask that researchers consider carefully whether exclusion of marginalized/minoritized populations is necessary or justified—particularly when NATIVE SPEAKER is used only as a way to achieve linguistic homogeneity. Instead, we urge psycholinguists to explicitly state the specific axes traditionally implied by NATIVENESS that they wish to target. We outline several of these (e.g., order of acquisition, allegiance, and comfort with providing intuitions) and give examples of how to recruit and describe participants while eschewing NATIVE SPEAKER. Shifting away from harmful conventions, such as NATIVE SPEAKER, will not only improve research design and analysis, but also is one way we can co-create a more just and inclusive field.

## Introduction

This article problematizes the use of NATIVE SPEAKER^1^ as a construct in language research. We argue that the concept is both vague and harmful, and advocate for the field of psycholinguistics to move forward with a more careful, considered, and nuanced view of language experience.^2^ We suggest that NATIVE SPEAKER is more accurately thought of as an ideology rather than an idealization, and give recommendations for how to shift our research practice accordingly.

The structure of the article is as follows. In Section “Introduction”, we present background on what the problems are, with regard to both vagueness and harm. Next, we connect these broader concerns with why it is an issue within psycholinguistics research, specifically relating to methodology and theory. In Section “Assessment and Implications of Current Practices”, we discuss implications of alternative approaches that could be taken in response to the big-picture issues detailed in Section “Introduction”, drawing from the literature in other disciplines that already problematize the concept. This section is divided into three stages of the research process: conceptualization, recruitment, task, and survey design, and data analysis. In Section “Actionable Recommendations”, we provide actionable recommendations for how psycholinguistic researchers can move away from NATIVE SPEAKER in their own work at each stage of the research process.

To represent a snapshot of the diversity of experiences that cannot be captured by NATIVE SPEAKER, we also provide four example profiles of language users that researchers may encounter (Boxes 1–4). We describe their language profiles (see the aspects of language experience laid out in “Complicating NATIVENESS in Recruitment, Tasks, and Surveys”) and return to them as examples throughout the paper.

### Vagueness

The term NATIVE SPEAKER is frequently used in language research. Perhaps precisely because of its frequent use and the assumption that it carries an intuitive meaning, the term is often not explicitly defined and operationalized. In some cases, where NATIVE SPEAKER is operationalized, the definition may be circular in nature and involve unstated, implicit assumptions. For example, according to Benmamoun et al. (2013), a NATIVE SPEAKER is someone that has “normal first language acquisition” (130) and that has “native” pronunciation. This description not only assumes that there are normal and abnormal acquisition processes (without detailing what those involve) but also refers to ‘nativeness’ in the definition to describe a criterion, ultimately failing to define the term NATIVE SPEAKER. Similarly, the antonym NON-NATIVE SPEAKER groups together an extremely heterogeneous set of individuals while strongly connoting a normative and monolingual experience. As Dewaele (2018b) puts it, NON-NATIVE SPEAKER is “inherently strange” as we are “defin[ing] somebody by what she or he is not” (236). If we take a closer look at the literature and compare definitions from various works, it is apparent that NATIVE SPEAKER is used more vaguely than one would imagine.

To exemplify the broad range of definitions for NATIVE SPEAKER, Table 1 lists some definitions of this term as used across linguistics and adjacent fields. Certain concepts come up frequently, but while some definitions associate NATIVE SPEAKER with multiple factors (e.g., Benmamoun et al., 2013), others associate NATIVENESS with just one factor (e.g., Boltokova, 2017). Importantly, while there is an array of definitions for NATIVE SPEAKER, there are many more papers that use the term without defining it. Drawing on these and other examples, several common trends in usage can be identified across the literature: *nativeness-as-history* (including age, order, and context of acquisition), *nativeness-as-proficiency* (including continued usage), and *nativeness-as-identity*. We briefly demonstrate how each of these themes—alone or in combination—are realized in the context of various sub-fields of language research.

**Table 1:** Some definitions of NATIVE SPEAKER.

Source	Definition of NATIVE SPEAKER	Facet(s) of language experience represented
Stern, 1983	“Native speakers have (a) a subconscious knowledge of rules, (b) an intuitive grasp of meanings, (c) the ability to communicate within social settings, (d) a range of language skills, and (e) creativity of language use.” (154)	Proficiency
Abrahamsson and Hyltenstam, 2009	A native speaker “(a) has spoken only Swedish at home during childhood; (b) has had Swedish as the only language of instruction at school; and (c) has lived his or her whole life in a context in which Swedish has been the majority language” (264)	History
Debenport, 2011	Speakerhood-as-identity: “Tribal members who play significant religious or political roles are more likely to be counted by San Antonians as ‘speakers’” (90)	Identity
Benmamoun et al., 2013	“A prototypical (educated) native speaker lives in a monolingual environment, or in a bilingual environment in which his/her original native language has not undergone attrition. Such a prototypical speaker is expected to have “native” pronunciation and a sizable, comprehensive vocabulary (about 20,000 words)” (130)	History and Proficiency
Rothman and Treffers-Daller, 2014	“A native language is one that is acquired from naturalistic exposure, in early childhood and in an authentic social context/speech community” (95) “Native speaker (i) bi-/multilinguals have multiple native languages; and (ii) nativeness can be applicable to a state of linguistic knowledge that is characterized by significant differences to the monolingual baseline.”	History

Linguistics as a field has historically conceptualized the NATIVE SPEAKER to be based on *proficiency* gained from a very specific “ideal” upbringing: They are the “ideal speaker-listener” with full mastery of a particular language and therefore able to provide authoritative judgments about grammaticality for any aspect of grammar (Chomsky, 1957, 1965). Chomsky’s (1965) description of NATIVE SPEAKER also implies that a speaker’s acquisition *history* must be monolingual^3^ in nature (such as the speaker profiled in Box 1), and this implication has been used as an underlying assumption in subsequent research (e.g., Sorace, 2004; Thráinsson, 2012; Benmamoun et al., 2013).

Box 1 **An example profile of a primarily monolingual speaker**

Ingrid, 65, grew up in The Netherlands and attended Dutch-medium schools. She learned Dutch from Dutch-speaking parents and spoke only Dutch at home, with friends, and in society at large. She learned written English and German in school, from ages 8–15, but does not use those languages in her daily life. While she can read in both languages, she isn’t comfortable speaking in either of them.

The written Dutch that she learned in school varies slightly from the spoken variety that she uses in her daily life at work. She is an avid reader of novels in Dutch and reads some news articles in English and German. She watches television and movies in Dutch and English, and uses Dutch subtitles for English media.

She considers herself to be a ‘native’ or ‘mother tongue’ speaker of Dutch.

In line with this, researchers studying second language acquisition or bilingualism have been known to assume and employ NATIVE SPEAKER or LANGUAGE to mean a high (or the highest possible) degree of *proficiency*. In this way, it is commonly used as a benchmark or comparison group for language learners or those who are considered otherwise “non-native” (e.g., Au et al., 2002; Abrahamsson and Hyltenstam, 2009). However, for the purposes of operationalization, many studies rely on acquisition *history* to identify native speakers. For example, Abrahamsson and Hyltenstam (2009) recruited ‘native speakers of Swedish’ based on language history (see Table 1). While comparing “native” Swedish speakers to reportedly “native-like” but “non-native” Swedish speakers, they assert that nativeness is a binary phenomenon like “‘marriedness’ and ‘deadness’” (267). The authors imply that there exists some threshold of language ability available to ‘native speakers’ that ‘non-native speakers’ cannot reach, as a result of age of language onset.

In other contexts, though ‘nativeness’ may still be taken for granted, the traditional definition of NATIVE SPEAKER breaks down. In sign language research, for example, the concept of a NATIVE SIGNER has proven to be rather elusive. This stems from the fact that signing individuals’ experiences are highly heterogeneous and idiosyncratic, differing in a number of ways from normative spoken language experiences (Quer and Steinbach, 2019). The strict definition of NATIVE SIGNER, based on language *history*, would include only second-generation deaf signers—that is, deaf individuals growing up with (deaf) signing parents.^4^ Since this is extremely uncommon, the idea of nativeness has been operationalized *via* a variety of criteria in sign language linguistics (e.g., Neidle et al., 2000; Costello et al., 2008; Mathur and Rathmann, 2009), involving aspects of *history*, *proficiency*, and *identity*. These have included some of the following: family environment (e.g., having deaf signing parents), early experience (e.g., prior to age 3), continued exposure and usage (e.g., daily contact), some indication of grammatical competence (e.g., ease of making judgments), and identification with the Deaf community.

In the context of immigration and language contact situations, continued usage (with links to *proficiency*) has also been implicated as a part of the definition of a NATIVE SPEAKER. Benmamoun et al. (2013) define “language attrition” as “the [gradual] loss of aspects of a native language by a healthy native speaker,” going on to say that “a native speaker will become, in the judgment of his or her peers, a non-native speaker of his/her own language” (132). This suggests that the status of NATIVE SPEAKER in this conception relies on degree of language use and maintained language ability.

Finally, though much of the previously outlined usages of NATIVE SPEAKER/SIGNER appeals to, or at least brings with it an assumption of, competence (cf., Dewaele, 2018b), not all definitions do. Rothman and Treffers-Daller (2014), for example, argue for the inclusion of heritage bilinguals (see example profile in Box 2) under the umbrella of NATIVE SPEAKERS (see Table 1). This definition is similar to the sign language research context in prioritizing early naturalistic exposure (*history*), but explicitly excludes the expectation of *proficiency* at any level.

Likewise, in contexts of language reclamation, being a (NATIVE) SPEAKER of a language does not come with connotations of proficiency at all, but instead is used in the sense of *identity* and membership. In the case of the Dene Tha community in Chateh, located in Alberta, Canada, among young people, “there is a strong self-identification with one’s heritage language and culture and a deeply rooted personal belief in belonging, as full and rightful members, to this language community” (Boltokova, 2017, p. 22). For communities like this one (cf., Debenport, 2011 who discusses a Pueblo community in San Antonio), ‘native speaker’ only refers to identity. In this way, the Dene Tha youth consider themselves as ‘native speakers’ without necessarily speaking the language with high fluency—or as some would say, “native” proficiency.^5^

Box 2 **An example profile of a “heritage”/immigrant bilingual**

Amy, 23, was born in Hong Kong and lived there until age 2 when her family emigrated to Toronto, Canada. She was first exposed to and began speaking only Cantonese. After moving to Canada, she began to hear English *via* immersion in a preschool setting starting at age 3, but still spoke only Cantonese otherwise.

After starting elementary school around age 5, Amy began to spend increasingly more time exposed to English. She continued to hear and speak Cantonese at home with family (including some media like TV and songs), with a couple of family friends, at certain extracurriculars (e.g., Saturday school for Cantonese) and in some places in the community that she went to with family (e.g., church, restaurants, grocery stores). Otherwise, English was heard and spoken at school, with peers and friends outside of school, at most extracurricular activities (e.g., sports teams, volunteering/work) and most places in the community. During this time, she gradually became less comfortable using Cantonese to communicate.

Currently, Amy uses English for almost everything other than speaking to her parents. She considers herself to not be fluent in speaking or listening to Cantonese. Due to some years of Saturday school as a child, she can read and write a small amount. Based on this, she considers English as her dominant, strongest and effectively only language. She doesn’t fully identify as a native speaker of either language, but she would say Cantonse is her mother tongue, while she speaks more like a (near-)native speaker of English.

As we can see, there is no clear, consensus definition of NATIVE SPEAKER/SIGNER or NATIVE LANGUAGE. This multifaceted concept can, but does not always, involve a constellation of factors relating to age, order, and context of acquisition (e.g., Costello et al., 2008; Abrahamsson and Hyltenstam, 2009; Rothman and Treffers-Daller, 2014; Quer and Steinbach, 2019), continued usage and/or exposure (e.g., Costello et al., 2008; Benmamoun et al., 2013), proficiency or competence (e.g., Chomsky, 1965; Abrahamsson and Hyltenstam, 2009), and sociocultural identification or membership (e.g., Debenport, 2011; Benmamoun et al., 2013; Boltokova, 2017).

Not only do researchers include different combinations of the above factors in their definition, the specifications of each criterion can also vary. Proficiency, for instance, is sometimes assumed, sometimes measured in a certain domain *via* assessments or tasks, and sometimes related to dominance, where the “strongest” language is considered the native language, rather than some measure of “absolute” level of proficiency. Age of acquisition, while often invoked, varies as to the exact ages that matter, for example, birth (Johnson and Newport, 1989) or age 3 (e.g., Costello et al., 2008; Dewaele, 2018b). Throughout many of these uses, the term is associated with assumptions of monolingualism and acquisition as a first language in contexts where there are clear temporal orders to learning different languages (Cook, 1999; Benmamoun et al., 2013; Dewaele, 2018b). All this together suggests that NATIVE SPEAKER, when used, can and does refer to disparate aspects of language experience across fields, studies, and contexts. We argue that the vagueness of this term is one reason to reconsider the extent to which NATIVE SPEAKER/SIGNER is a relevant and useful concept in our research.

### Harm

All conceptual categories used for research are inherently simplifications and cannot capture the complexity of social life, but are necessary because there is no way to conduct meaningful research without them. However, a sensible scientific aim can be to ensure that the terms we use both describe the phenomenon of interest as accurately as possible and do not harm the communities we study. The term NATIVE SPEAKER meets neither requirement: (i) As argued in the previous section, it is ambiguous and thus a hindrance to data analysis and rigorous theory construction, and (ii) as we argue in this section, it can be harmful, particularly to minoritized individuals and groups, in that use of NATIVE SPEAKER in academic research reproduces normative assumptions about linguistic behavior, experience, and identity.

As illustrated in Section “Vagueness”, the range of its use in research implies that the NATIVE SPEAKER is an “ideal speaker listener” (Chomsky, 1965) who has had a particular acquisition experience (learning one named language in childhood in a linguistically homogeneous environment before learning other languages); who is “highly proficient” in one named language; who has continued to use the same named language from childhood to adulthood; and for whom that language is part of their sociocultural identity. Crucially, this does not take into account the fact that a single named language cannot fulfill all of these roles for most individuals and communities around the world due to structural factors, such as globalization, colonialism, ableism, and linguistic discrimination of various types (cf. Ortega, 2020).

These normative assumptions of NATIVE SPEAKER reinforce hegemonic conceptions of language use, ability, acquisition, and linguistic identity. When researchers use NATIVE SPEAKER in their work, and when participants are excluded from research because they do not fit researcher expectations of a NATIVE SPEAKER, they perpetuate deficit perspectives toward multilingualism and non-hegemonic modes of language acquisition. This can (perhaps, inadvertently) frame these individuals and their practices as deviating from the norm, thus contributing to racialized conceptions of “nativeness” and feelings of LANGUAGELESSNESS among those whose speech is positioned as abnormal, in which individuals might be categorized (either by themselves or others) as not speaking any language at all (Rosa, 2016; Ramjattan, 2019). The linguistic experience of most of humanity does not conform to these assumptions, making the term both widely inapplicable and harmful, as it leads to the systematic exclusion of marginalized populations and perpetuation of deficit perspectives.

To return to an example from Section “Vagueness”, most conceptions of NATIVE SPEAKER exclude the overwhelming majority of signers from being considered as “native” for research purposes. According to Quer and Steinbach (2019), most deaf children are not raised in environments “where there is *adequate sign language input* for the child to develop language competence *in a natural way*” and “do not fall under the strict definition of native speakers or signers” (2, emphasis our own). Limiting sign language research to only include deaf children born to deaf adults would not be representative of the use of signed languages in the world (see example in Box 3), and it is moreover harmful to position the acquisition contexts of the majority of signers as being inadequate, especially without attending to the structural reasons for this (i.e., Oralism and other forms of ableism).

Box 3 **An example profile of a mobile Deaf signer**

Angel, 38, is deaf and was born in Manila, Philippines. She moved to San Francisco, CA, in her 30s and now lives in Boston, MA, with her wife, who is also deaf. Growing up, she spoke/signed Tagalog, English, and Filipino Sign Language at home. In school, she used Tagalog and English, as she was integrated into a class of hearing students (“mainstreamed”) on her own, with no interpreters or special support. Now, she primarily uses English and American Sign Language (ASL), and considers English to be her strongest language.

Angel attended school from the age of 5; in the 1980s and 1990s there was no organized educational interpreting system in the Philippines, so she was immersed in a spoken language environment. Some of her family is deaf, and they primarily communicate in Filipino Sign Language. At home, her family also used Tagalog, English, Bisaya, and Hokkien. Angel took courses in Nihongo (Japanese) at a language institute and took online courses in Japanese Sign Language, which she signs with a few friends. In college, she took a Castilian Spanish course. She learned ASL from her wife and YouTube videos, as well as from interactions in Deaf spaces in the United States.

In informal settings, Angel is most comfortable speaking Taglish (code-mixing of Tagalog and English), followed by ASL. ASL is the language that she uses the most with her wife, kids, friends, and coworkers at the university where she works. At home, she uses ASL, English, and Tagalog. At work/school, she uses ASL and English. She uses ASL with her friends. Angel uses English with strangers, but uses ASL if the stranger happens to know it. She considers herself a native speaker/signer of Tagalog, English, and Filipino Sign Language.

Bucholtz (2003) discusses how the concern for “real language” and “authenticity” has made monolingualism appear unmarked in sociolinguistics. In the ideology of linguistic isolationism, research is based on the assumption that “the most authentic speaker belongs to a well-defined, static, and relatively homogenous social grouping that is closed to the outside” (404) and that “bilingualism and multilingualism are […] special rather than typical sociolinguistic situations” (405). Again, by positioning these linguistic experiences as abnormal and inauthentic, such frames position individuals themselves as abnormal and inauthentic.

In English Language Teaching (ELT), NATIVE SPEAKERISM and its associated harm have been deeply theorized, with Holliday (2006) arguing that this term represents an ideology that ‘native speakers’ are better equipped to teach English than ‘non-native speakers.’ Scholars who have expanded upon this work have shown that who is seen as a NATIVE SPEAKER of English is racialized, and prizing the speech and labor of perceived NATIVE SPEAKERS of English also ends up prizing whiteness (Gerald, 2020). In the context of Canada, Ramjattan (2019) shows how White ‘native speakers’ are perceived as being better teachers and more qualified, and, even beyond the context of North America, White speakers of English are more likely to be perceived as “native” (Sung, 2011; Lee and Jenks, 2019).

While many studies connecting race and nativeness are situated in the context of ELT, the ideologies that are described are certainly not limited to these contexts. Rosa and Flores (2017) discuss how “unaccented English” is conceptualized by English users as an English which conforms to White listeners’ expectations. Rubin (1992) and following studies (Babel and Russell, 2015; Kutlu, 2020; et alia) using a matched guise paradigm have shown repeatedly that recordings played alongside White faces are rated as “more native,” “more intelligible,” or “less accented” than non-White faces. These ideologies are present in the world, and there is an opportunity for language research practitioners who want to create a more inclusive discipline to denaturalize the often implicitly made connections between ‘nativeness’ and race. This can be done by accounting for the possibility that our participants may hold these ideologies, as well as accounting for the possibility that we as researchers may also hold these ideologies, which can lead to systematic exclusion of racialized individuals from research.

Monolingualism as the norm is often implied in the term NATIVE SPEAKER (see “Vagueness”), and such assumptions have harmful consequences for multilingual (or multilectal) individuals, especially those who are racialized. Rosa (2016) discusses how deficit perspectives are employed when talking about the natural multilingual practices of racialized individuals, such as language mixing. Rosa shows how languagelessness is assigned to racialized individuals who enact non-normative language practices; these individuals are labeled as not speaking any language at all. This feeling of languagelessness is also ascribed by multilingual individuals to themselves and is certainly not unique to the Global North; in the context of South India, Namboodiripad (2021) showed that Malayalam speakers for whom Malayalam was their first and most-used language felt that, because they mixed languages, they were not able to speak any language at all (see also Box 4). Across contexts, not conforming to a monoglot norm can make speakers themselves feel deficient, and institutional sites of language evaluation, whether in schools or in psycholinguistics experiments, can reinforce these negative and harmful ideologies.

Box 4 **An example profile of a multilingual individual experiencing globalization**

Leela, 27, was born in Kerala, India. The first language she was exposed to and spoke was a high contact variety of Malayalam, including elements from Tamil, Hindi, and English. She attended English immersion school, starting at age 4. While she learned to read and write Malayalam in school, Malayalam language classes ended at age 12. She also learned to read and write Hindi in school from ages 8–12.

Growing up, she heard and spoke Malayalam mostly at home with her family and in the community at large. She also heard and spoke English and Malayalam in school with peers and friends. She consumed mostly Malayalam and English media, but also sometimes watched Hindi and Tamil movies. Currently, she uses a high-contact variety of Malayalam at home, in social settings, and with greater society. She uses Malayalam and English at work (English with clients, superiors, and for all written communications; Malayalam with friends and in casual conversations); English when traveling outside of India; and English and Hindi when visiting family in North India. She also watches Hindi, Malayalam, English, and Tamil movies.

She doesn’t consider herself fluent in Malayalam both because she prefers to read and write in English (though she can read and write in Malayalam), and because she doesn’t feel like she can speak Malayalam without using English elements, especially depending on the semantic domain. She considers Malayalam her Mother Tongue, which is a locally relevant term, but states that she doesn’t feel proficient in any language.

The harm in creating a context in which languagelessness is imputed to (particularly racialized) multilinguals must be understood in a historical context in which ascribing languagelessness has been a tool for dehumanization. Degraff (2005) discusses how delegitimizing the languagehood of creoles was used as a tool of colonialism and chattel slavery, and how it led to dehumanizing the people who were being oppressed. DeGraff points out the continuity of this dehumanization in how creoles are described as exceptional languages that are birthed from “imperfect learning,” and connects this to colonial narratives about how creoles were the result of “a race that is linguistically inferior” trying to learn colonial languages (Vinson, 1889, cited in Degraff, 2005). Looking at discourses on African American Language(s), we see similar deficit perspectives which are based on essentialist ideas about language attainment and learning (e.g., Green, 2004 on “dual components” approaches). Constructs, such as NATIVE SPEAKER, carry with them essentialist and harmful ideas about language and linguistic attainment, and psycholinguists who would like to push against such harm should reject and work against narratives which dehumanize our participants, our colleagues, and ourselves.

### Connections to Research Methods and Theory

A well-designed experiment is detailed, makes clear predictions, targets a specific population, and involves a data analysis plan. Given this, the vague and harmful definitions of NATIVE SPEAKER pose significant methodological problems for the field of psycholinguistics.^6^ As evidenced in the sections above, researchers make various implicit assumptions about the language experience of a NATIVE SPEAKER and these assumptions shape all aspects of research, ranging from question creation to data analysis. We make explicit these connections in this section, focusing on research conceptualization and design followed by comparison groups and analysis. As we will see, using NATIVE SPEAKER can lead to imprecise predictions, ill-selected samples, exclusionary and inconsistently defined participant pools, inappropriate materials, and misguided analyses.

#### Issues With NATIVE SPEAKER at the Stages of Conceptualization and Design

Some psycholinguistic research deals with specific predictions about how “native” and “non-native” speakers process or produce language (e.g., production of phonetic variability; Baese-Berk and Morrill, 2015; Vaughn et al., 2019; see also Bosker et al., 2014 on hesitation phenomena) or how different listeners process or perceive “native” or “non-native” (-sounding) language (e.g., comprehension, perceptual adaptation, or credibility of “foreign-accented” speech; Lev-Ari and Keysar, 2010; Hanulíková et al., 2012; Baese-Berk et al., 2013; Lev-Ari, 2015; Bent et al., 2016). However, even when NATIVENESS is not central to their research questions, researchers tend to recruit a sample of ‘native speakers’ for participation or for stimuli development. From a superficial search of research in top psycholinguistics journals, we found some examples which illustrate how this often looks in psycholinguistics research. Here is one pair of examples which covers participants: “Thirty native speakers of English from the University of York student community took part in this study” (Altmann, 2004), and “Twenty-four University of Rochester undergraduates who were native speakers of American English … were paid $10” (Kurumada et al., 2014). In the same papers, we found examples of how those selected to create stimuli are often described: “The sentences were recorded by a male native speaker of British English” (Altmann, 2004), and “A native speaker of American English recorded two tokens of each item” (Kurumada et al., 2014).^7^

As illustrated in Section “Vagueness”, NATIVE SPEAKER varies in definition across researchers and often goes completely undefined. One problem with this is that the researcher’s idea of who a NATIVE SPEAKER is may not match the participant’s idea of who a NATIVE SPEAKER is, if the concept is even clear to the participant. Faez (2011), for instance, presents several case studies that illustrate how (i) participants can have difficulty answering the question of whether they are “native” and (ii) an individual’s self-ascribed “native” status may not be matched by the judgments of outside informants (nor do judges always agree with each other). This can lead to a discrepancy between the researcher’s target sample and the actual individuals recruited. Another consequence is that different laboratories may be using the term inconsistently, which is problematic for replications, follow-up studies, and cross-study comparisons. For example, NATIVE SPEAKER study inclusion criteria commonly exclude the so-called “heritage speakers,” such as Amy (Box 2), from participation—often based on harmful deficit perspectives—but some do not. Moreover, the extent of social and linguistic variation across those who are identified as ‘native speakers’ may not be fully considered by researchers. In this case, the inclusion of imprecisely defined ‘native speakers’ in stimuli norming or creation may result in biased stimuli, or stimuli that are inappropriate for the target participant demographic and research question (e.g., the regional variety spoken by a speaker recording audio stimuli or judging acceptability for stimuli may differ compared to the participants in the experiment).

Simply reporting that ‘native speakers’ participated or recorded stimuli clearly does not provide information adequate for replication. These issues are especially concerning given the replication crisis plaguing psychological research (Open Science Collaboration, 2015; Camerer et al., 2018). Henrich et al. (2010) noted that narrow samples from Western, Educated, Industrialized, Rich, and Democratic (WEIRD) populations are frequently used to make broad claims about human psychology and behavior.^8^ So, too, linguists have traditionally used monolingual speakers from relatively homogenous (and WEIRD) speech communities as a baseline to make broad claims about language organization and behavior (Evans and Levinson, 2009; Croft, 2013; Dahl, 2015; see also Sedarous and Namboodiripad, 2021 on the overrepresentation of Written, Institutionally supported, Standardized, and Prestigious (WISPy) languages in psycholinguistics). It is important to recognize that such speakers are exceptional, rather than the default case. The centering of binary, essentialist conceptualizations of native language competence leads to the exclusion of minoritized communities from research, as described in Section “Harm”.

In addition, the implicit assumptions underlying NATIVE SPEAKER inherently espouse certain theoretical frameworks. For example, many researchers cite that learning a language at a young age is a key aspect of what defines a native speaker (e.g., Cook, 1999; Costello et al., 2008; Rothman and Treffers-Daller, 2014; Hall et al., 2017) and anyone who learns a language after this arbitrary cutoff (see discussion in “Vagueness”) is no longer a NATIVE SPEAKER. While the existence of a biological critical (or sensitive) period is debated and not all language researchers agree with the idea (see, for example, Schouten, 2009; Balari and Lorenzo, 2015), this is precisely the theory that researchers implicitly adopt when they use NATIVE SPEAKER as a proxy for “someone who learned a language at a young age.” For researchers who do not agree with the idea of a critical period, invoking this theoretical framework when they use NATIVE SPEAKER can be an issue. Researchers should be aware that the way that they define NATIVE SPEAKER may invoke theoretical frameworks that they do not necessarily agree with.

Inconsistent and vague definitions of NATIVE SPEAKER also pose issues for theory construction. By building solely upon conclusions from studies that inconsistently define their variables, use differing methods to categorize participants, and analyze participants based on these groupings (see “Issues with NATIVE SPEAKER When Constructing Comparison Groups and Conducting Analyses”), we will invariably create theories that fall prey to these same issues. In addition, the hegemony of research conducted in contexts where monolingualism/normative language use is seen as a control or neutral mode structures our fields of inquiry such that multilingualism/non-normative language use is peripheralized, requiring extra theoretical and methodological machinery. This privileges and incentivizes the study of certain, dominant groups over others, and puts research on socially less-powerful groups at an inherent disadvantage. Taking NATIVE SPEAKERS out of the center of our fields will not only sharpen research questions, but also expand the types of research that is done.

#### Issues With NATIVE SPEAKER When Constructing Comparison Groups and Conducting Analyses

When operationalizing concepts, psycholinguists often categorize participants into groups for the purposes of comparison, either *a priori* or in *post-hoc* examination of the collected data. Groups can include NATIVE SPEAKER, NON-NATIVE SPEAKER, L2 LEARNER, HERITAGE SPEAKER, and countless others. While many researchers use dimensions of language experience, such as age of acquisition, order of acquisition, or continued exposure, often collected *via* language experience questionnaires, others use proficiency tasks to group participants (e.g., picture naming, cloze tasks, and standardized language tests).

These different methods and criteria lead to huge variation in how participants are categorized and treated in analyses across studies. While the use of standardized measures or tasks may appear more objective or consistent, different assessments can often categorize participants in significantly different ways (Solís-Barroso and Stefanich, 2019). This poses a crucial question for the field of psycholinguistics: Are the groups in language experiments comparable across studies? Further, researchers’ constructed categories contain their assumptions about the term (NON-)NATIVE SPEAKER and ignore the ways in which these artificially “different” groups could be similar in their perception and production of language (for some work that problematizes this assumption, see Dąbrowska, 2013; Han et al., 2016; Johns et al., 2018). These categories are used in analyses to make broad generalizations about diverse and ill-defined groups of speakers which may not apply across different segments of the “same” population.

Of course, individual differences will always exist in any sample. However, when using underspecified categories, such as (NON-)NATIVE SPEAKER, there may also exist large systematic “within-group” differences that can affect the linguistic variables of interest, and therefore complicate theoretical interpretation and generalization. Because we often assume homogeneity in such groups, potentially relevant factors of language experience are inconsistently reported. For example, Surrain and Luk (2019) found in their review of research comparing bilinguals to monolinguals that there were systematic regional differences in what types of information was collected, with sociolinguistic context being about three times as likely to be reported when taking place outside of North America and Europe. If these types of linguistic experience are not collected or reported, we may not know the extent to which different samples are comparable.

To take one example, MONOLINGUAL NATIVE SPEAKERS in fact vary immensely in experience with other languages or varieties; though researchers do not always take this into account, it can lead to significant differences in linguistic behavior. For instance, lifetime experience with other speech varieties (e.g., living in an urban metropolis vs. small rural town) influences comprehension (Laturnus, 2018), while even short periods of exposure to another language can affect “native language” speech production (e.g., Chang, 2012). In addition, Dewaele (2018a) discusses how exposure to British English caused semantic restructuring in individuals who had grown up using American English. It behooves us to remember that the difference between a language and a variety is gradient and socially constructed, and, as such, even “Native Speakers of American English” represent a highly heterogeneous population whose particular language histories are likely relevant for various psycholinguistic processes.

These issues relate to a noted historical tendency of “categorical thinking” in psychology and psycholinguistics *via* an overreliance on factorial design and treating continuous predictors as categorical in analysis. The risks of discretizing continuous measures for analysis and the benefit—or indeed, necessity—of maintaining these continuous measures have also been argued for language research on conceptual and empirical grounds (see MacCallum et al., 2002; Baayen, 2004; Balota et al., 2004; Young, 2016). Categorizing continuous variables can not only lead to a decrease in statistical power, increased potential for spurious finding, and a reduced ability to detect complex and/or non-linear relationships (see Cohen, 1983; Young, 2016), but also have more general implications for interpretation and theory formation.

These problems are directly relevant to the case of experimental linguistic research where measurement of ‘nativeness’ and associated concepts (e.g., bilingualism and language dominance) has been historically inconsistent across the literature and often relies on binary categorization of continuous variables (e.g., Solís-Barroso and Stefanich, 2019; Ortega, 2020). Solís-Barroso and Stefanich (2019) show through the comparison of different measures of language dominance that treating language dominance as a categorical variable is problematic, given that an individual bilingual will not be consistently placed into the same dominance group depending on which assessment is given, contributing to potential heterogeneity within each group. By moving away from categorical thinking when it comes to participants, we allow for the discovery of more precise factors which influence language understanding and use.

## Assessment and Implications of Current Practices

In this section, we discuss possible responses to the problems outlined in Section “Introduction”, drawing from the approaches taken by researchers from various fields. These are organized into three (broadly defined) stages of research: conceptualization, recruitment, task, and survey design, and data analysis. We review the effectiveness of these solutions, along with representative examples, leading into our more specific recommendations in Section “Actionable Recommendations”.

### Complicating NATIVENESS in Conceptualization

Although scholars have suggested both narrowing (e.g., Cook, 1999) or broadening (e.g., Rothman and Treffers-Daller, 2014) the scope of the definition of NATIVE SPEAKER, others have argued to leave behind (NON-)NATIVE SPEAKER (and “mother tongue”) in favor of more specified characterizations. For example, Rampton (1990) recommended decomposing NATIVE SPEAKER into (i) language *expertise* (linguistic knowledge and ability) and (ii) language *loyalty/allegiance* (social identification which can both be gained *via inheritance* or *affiliation*). These alternatives allow us to conceptualize two different facets (analogous to *proficiency* and *identity*) without appealing to NATIVENESS. For example, Amy (Box 2), a “heritage” bilingual, might consider herself an *expert* in English with less *expertise* in Cantonese. At the same time, she holds *allegiance* to both Cantonese (*via inheritance*) and English (*via affiliation*). This distinction on its own, however, does not specifically account for aspects of linguistic *history*.

Dewaele (2018b) proposed that we replace NATIVE and NON-NATIVE SPEAKER labels with L1 and LX (e.g., L2, L3, and L4) in an effort to more specifically represent language history (i.e., early experience) separate from aspects of proficiency and identity. Thus, a researcher with specific hypotheses about individuals who learned a language earlier or later in life could compare them without drawing on NATIVENESS. For Amy (Box 2), Cantonese would be her L1, while English could be considered an LX—however, Dewaele’s proposed cutoff for counting a language as LX is “after the age of 3years” which may designate English as a second L1 for Amy. Nevertheless, while this terminology gets away from the hegemonic associations of NATIVE vs. NON-NATIVE, it still contains assumptions of normative ordered acquisition, which is not the case in many multilingual or globalized communities, and a critical period effect, which carries with it many other theory-specific assumptions. For Angel (Box 3), there are several possible L1s, but many of them are not very relevant for her language use across the bulk of her lifespan. These examples demonstrate how labels, such as L1 and LX, require researchers to rely on categories that may not be well-motivated, a practice that comes with many disadvantages (see “Complicating NATIVENESS in Data Processing and Analysis” and “Alternatives to NATIVE SPEAKER in Data Processing and Analysis” on Continuous Variables).

Regardless of the alternative terminology one chooses to use as labels or descriptors, we believe the best practice is to use specific characterizations of particular aspects of language experience (e.g., proficiency, history, and identity). This aligns with recommendations within bilingualism research to increase comparability across laboratories and studies by “provid[ing] detailed descriptions of the populations tested following a consistent approach” (Marian and Hayakawa, 2021, p. 7). By avoiding the conceptualization of (NON-)NATIVE SPEAKER at all levels of research, we are able to simultaneously (i) clarify our theoretical stance and interpretations, (ii) reject normative assumptions of the background of (NON-)NATIVE SPEAKERS, and (iii) acknowledge the heterogeneity of linguistic knowledge and behavior, even among those with purportedly similar backgrounds.

As discussed in Section “Issues With NATIVE SPEAKER When Constructing Comparison Groups and Conducting Analyses”, even within supposedly MONOLINGUAL NATIVE SPEAKER populations from the same region, literacy and education can vary extensively, not to mention diversity in experience with other languages or language varieties. For example, Ingrid (Box 1) is relatively well-read and thus has consistent exposure to the types of syntactic structures disproportionately represented in written Dutch, such as sentences with multiple embeddings (see van der Wouden et al., 2002). However, another self-identified Dutch monolingual may not read much literature and therefore have a meaningfully different amount of exposure to multiple-embedded constructions. These factors, if not taken into consideration by the researcher, could lead to groups that are overly heterogeneous or otherwise not well-controlled. Clearly, delineating the relevant and irrelevant characteristics for the target research sample would allow for better control over homogeneity (if homogeneity is, indeed, the goal of the researcher—see “Harm” and “Issues With NATIVE SPEAKER at the Stages of Conceptualization and Design” for reasons why this might not be a desirable goal), as well as ensuring that groups used for purposes of comparison are indeed comparable or contrastive on the dimensions of interest to the researcher.

An additional benefit is that this practice, through increasing deliberate and thoughtful development of inclusion and exclusion criteria, can help to minimize the exclusion of underrepresented groups or individuals who do not fit into normative assumptions, but may in fact match the criteria for a particular research sample. This also potentially expands our participant pool, which may provide the practical advantage of facilitating research recruitment. In a similar vein, although a common practice in psycholinguistic research is the recruitment of ‘native speakers’ to norm or judge stimuli, or ‘native, monolingual speakers’ to record auditory stimuli, we cannot assume that these individuals have “neutral” identities or normative language histories. The same careful consideration of relevant and irrelevant characteristics of individuals under study should be applied to perceptual judges and speakers for stimuli.

### Complicating NATIVENESS in Recruitment, Tasks, and Surveys

As researchers who have employed a critical approach to understanding research methods in linguistics and related fields have shown, it is imperative to understand how the questions we ask and the ways we ask them will be interpreted in local contexts, because these may differ significantly from researchers’ expectations (Briggs, 1986; Hill, 2006). All research with human subjects, even research on language itself, constitutes a communicative event between research participants and researchers. Coming from a background in the academy, we have certain expectations about the communicative event that constitutes research, and the accepted norms of this event. However, our participants often do not come from a background of institutionalized research agendas and have differing familiarities with the communicative routines used (from survey, interview, and elicitation, to experimental task) and therefore do not share the same expectations for how the communicative event should unfold. Particularly, using the term “native” in recruitment may be understood differently by participants depending on their backgrounds, the context, and their understandings of research. In addition, translations of the term “native” could be interpreted in ways that researchers did not intend when designing the study, and unintentionally include or exclude participants.

As an example, a study of Hindi-Urdu sentence acceptability (Upreti and Namboodiripad, in prep.) asked a range of questions about exposure, comfort, and use of Hindi-Urdu. They also asked participants, at the end of the language experience survey, to indicate if they consider themselves “native speakers” of (a) Hindi-Urdu and (b) English. There were several cases in which participants’ self-identification as “native speaker” did not align with many commonly understood correlates of nativeness: One participant had grown up in Pakistan (where Urdu is an official language) and spent the first 35years of his life in regions where Hindi-Urdu were the dominant languages. English and Hindi-Urdu were among the languages he heard growing up, and these were the two languages of instruction in his schooling. He rated himself as being maximally comfortable in reading, writing, listening to, and speaking Hindi-Urdu. And yet, he selected “no” when asked if he considered himself a native speaker of Hindi-Urdu (he also selected “no” for English, the language he considers his strongest). If that question had been the one used to recruit participants, this person would not have opted in, despite the fact that his language experience, as measured by the other questions, is highly relevant for this study.

Conceptions of the term NATIVE SPEAKER vary widely depending on how it is translated and the local context of its use. In many cases, some variant of “native language” is used on government censuses, and understandings of the term are influenced by historical uses and its connection to this survey. For example, in post-Soviet states, Soviet language planning tied languages to national territories, called Soviet Socialist Republics (SSRs), within the USSR. Language became a salient marker of national identity linked to the ability to find employment and access to resources in SSRs, and it was beneficial for people to identify their native language as that of the titular nationality (Slezkine, 1994; Martin, 2017). In the present, ties between native language and nationality still exist, and thus, asking for someone’s “native language” will most likely elicit a response coinciding with their identity (e.g., Kyrgyz language, Kyrgyz, Kyrgyzstan) even if they speak primarily Russian, feel most comfortable speaking Russian, and learned Russian in early childhood. Thus, it is important during recruitment to ask tailored, specific questions about language use and experience to recruit appropriate participants, rather than asking whether they are a NATIVE SPEAKER of a language.

We also must consider that translations are not one-to-one equivalents of meaning, and the categories of NATIVE SPEAKER and its translations, though they will most likely overlap somewhat with understandings of the term in English, will not match completely. The common translation of “native language” in Russian, “rodnoĭ iazik [родной язык],” comes from the root “rod [род]” with associations of “birth” or “tribe” or “natural” and is found in words, like parents (roditeli [родители]) and homeland/motherland (rodina [родина]; Patrick, 1989). A rough translation is more like “mother tongue” or “birth tongue,” but also carries connotations of national identity from its association with states (motherland) and historic uses in the census and language planning described above. As a consequence, a study asking for participants who are “native speakers of Russian” might recruit people who are from Russia, who identify as Russian, and whose parents or family speaks Russian but leave out those who believe Russian is the language they are most proficient in. Most problematically, asking this question could exclude the millions of non-Russians who use Russian, and misrepresent Russian as it is spoken by the majority of Russian-speakers in the world. Asking more targeted questions about the aspect of language use that is pertinent to the study, such as those outlined in Table 2, and understanding how these questions might be interpreted in local contexts will mitigate problems arising from unequivalent translations and conceptions of the term.

**Table 2:** Questions which can be used to probe various factors of language experience in survey design.

Factor of Interest	Categorical Questions (Useful for Recruitment)	Open-Ended/Gradient Questions (Useful for Survey Design/Capturing Continuous or Qualitative Differences)
Age and Order of Acquisition	Did you start learning [Language X] before [Age Y]	At what age did you begin learning [Language X]?
	Is [Language X] (one of) your first language(s)?	*Consider probing* How long was [Language X] used before exposure to another language?
Context of Acquisition	Did you grow up speaking [Language X] in [Region Z]?	Please list the locations you have lived in, the ages you lived there and the languages you spoke during that time.
	Did you grow up speaking [Language X] at home/in school?	What percentage of the time did you speak/hear [Language X] with your family growing up?
	Were you exposed to [Language X] at home/in school?	How many years were you exposed to [Language X] at home/in school?
	Was [Language X] the language of instruction in school?	How many years was [Language X] the language of instruction at school?
		*Consider probing* Presence of and/or interaction with [Language X]-speaking community networks
		Interest in [Language X] media
Language Proficiency/Usage Practices	Can you speak/read [Language X]? / Are you a (fluent) [Language X] speaker? / Are you comfortable speaking [Language X]?	Please rate on a scale of 1–7 your proficiency/fluency/comfort in [speaking/understanding/reading/writing; Language X].
	Do you mainly use [Language X] (at home/at work/in daily life)?	What percentage of the time do you speak/hear [Language X] at home/work/school/with friends?
		*Consider probing*
		Language use in different spheres (e.g., home, work, and school)
		Literacy
		Which language varieties they use
Language Identity/Allegiance	Are you a [Language X] speaker?	Please rate on a scale of 1–7 how much you agree with the following statements: “[Language X] is my [native language/Mother Tongue/etc.]”
	Do you consider yourself a [Language X] speaker?	“[Language X] is my preferred language”
	Do you consider [Language X] to be your? [contextually relevant term, e.g., native language, Mother Tongue]?	“I feel a strong connection to [Language X]”
		*Consider probing*
		Feelings or perceptions of pride, value, community, and nationality related to using or learning [Language X]

Given that people have different understandings of the term, particularly across sociolinguistic contexts, we note that merely asking participants to report whether they are ‘native speakers’—without explicitly stating how the term is defined—is also not effective for assessment. Additionally, multilingual speakers often tend to under-rate or be unsure of their own linguistic abilities as a result of negative discourse surrounding their speech communities. This may influence identity as a ‘native speaker’ and is especially relevant for multilinguals who might be racialized (Tomoschuk et al., 2019; Ortega, 2020; see also Gullifer et al., 2021 on context-based mismatches between “native” language proficiency self-ratings and objective task measures). We see these issues reflected in the example profiles (Boxes 1–4) where individuals’ self-characterization of their native language(s) varies and may not always align with researchers’ goals (see also case studies in Faez, 2011).

In a study comparing four language dominance measures, some of which are also used to test ‘nativeness,’ Solís-Barroso and Stefanich (2019) found that out of 29 Spanish/English bilinguals tested, 20 were categorized differently depending on which measure was used. That is, even if multiple assessments claim to measure the same factor (e.g., ‘nativeness,’ proficiency, or dominance), they do not always yield the same categorization of a participant. To illustrate, imagine that Measure A defines ‘nativeness’ as solely (1) being born in a household where that language is spoken. Separately, Measure B operationalizes ‘nativeness’ as (2) being highly proficient in a language and (3) having no detectable ‘accent.’ Finally, Measure C requires all three criteria be met to be considered “native.” In different studies, then, Amy (Box 2) could be considered “native” in Cantonese only (Measure A), “native” in English only (Measure B) or neither “native” in Cantonese nor English (Measure C). Note that these outcomes would not necessarily align with self-report either, which also may vary depending on whether she is asked about her first language, “mother tongue” or “native language.”

Further, it is important to highlight that individuals, especially multilingual speakers, have varied skill/comfort levels in different dimensions of language, such as syntax, vocabulary, and phonetics. Additional variation may arise across these dimensions depending on the method of measurement (e.g., picture naming tasks vs. measures of online processing; Birdsong, 2014). Moreover, both monolingual and multilingual individuals experience changes in use and proficiency across the lifespan, complicating the practice of assessing ‘nativeness’ while relying on a static measure in a single domain.

Leela (Box 3), for example, would likely perform “natively” in a Malayalam and English picture naming task, regardless of the comparison group. However, depending on the norms assumed by the researchers, she might not perform “natively” in a phonetic task in English, or a reading comprehension task in Malayalam, because of the contexts in which she learned both of those languages. In addition, Leela now uses solely English at work; when she was a child, she was almost never in English-only contexts. As such, any assessment of her comfort in using English would likely change significantly across her lifespan. Crucially, despite spending her whole life in a Malayalam-speaking region and having an education background that is quite common for people her age, she would show quite different performance across domains. This not only demonstrates the problems with prioritizing certain measures over others, but also could potentially lead to harm by reinforcing deficit perspectives toward multilingualism.

Potential histories of oppression and marginalization should also be accounted for. Surrain and Luk (2019) note that many standardized measures of language proficiency did not consider multilingual children in their norming process and that many of the standardized normed tests were developed to evaluate for atypical language behavior rather than proficiency. Care must be taken in the use and interpretation of standardized measures of language proficiency to avoid framing differences as deficits. Following Surrain and Luk, who found that only 38% of studies on children and 17% of studies on adults addressed sociolinguistic context in discussions of participants and results, we suggest that these details be reported in publications.

By being explicit about the aspects of language experience that we are concerned with, what assessments we use, and what assumptions our assessments carry, we can help avoid inconsistency. In the same vein, when selecting measures to characterize participants or while performing cross-study comparisons, we suggest researchers pay close attention to how ‘nativeness’ is defined and measured. Keep in mind that the fact that two studies use the term ‘native speaker’ does not guarantee that both studies share the same operationalization. The assessments chosen and how they are reformulated to be more targeted and specific will vary based on the relevant aspects of language experience that the researcher is interested in.

### Complicating NATIVENESS in Data Processing and Analysis

#### Continuous Variables and Linear Regression

Many scholars have noted issues surrounding entrenched “categorical thinking” in psychology and psycholinguistics, marked by sampling few values along a continuum, discretizing continuous measures, and an overreliance on factorial design and ANOVA (for more detailed discussion, see MacCallum et al., 2002; Baayen, 2004; Balota et al., 2004; Young, 2016). In some cases, categorizing continuous variables is purposeful or necessary and reflects a certain research goal; however, problems arise when this approach becomes entrenched and hinders theoretical progress. While dichotomizing continuous predictors generally results in a loss of power, Baayen (2004) notes that in the case where there is a single relevant predictor X, building a factorial contrast for extreme values of X (i.e., assigning the first 5% of ranked values to a “low” condition, and the last 5% to a “high” condition) can increase statistical power. However, this strategy comes at the price of being limited to making generalizations about the extreme ranges of X. A contrast between the high and low groups can be established, but no prediction is possible for the values of X in the intermediate range. Furthermore, as Balota et al. (2004) note, information about the amount of unique variance that a given factor accounts for in a given design should be taken into consideration in our theory formation and selection. Thus, Baayen (2004) recommends that “factorization is useful when obtaining data is costly and when documenting the existence of an effect is the sole purpose of the experiment” (6).

Scholars studying bilingual populations have argued for language proficiency, usage, and acquisition-related factors to be measured and analyzed gradiently. Luk and Bialystok (2013), for example, assert that “bilingualism is not a categorical variable,” finding that language proficiency and bilingual usage are two continuous factors which should be included in models characterizing bilinguals (see also Gullifer et al., 2021). Similarly, Ortega (2020), in a paper on heritage language development from a social justice perspective, argues that, in order to properly characterize the language of “heritage” language speakers (cf. Benmamoun et al., 2013), one must take a gradient approach to bilingualism. In addition, some sign language linguists have argued for the use of a continuous measure of nativeness, as opposed to a binary categorical definition (Costello et al., 2008). Treating a predictor, like “language dominance” as a continuous variable in analysis, allows for a more fine-grained analysis which answers questions about whether the more dominant a bilingual is in a given language, the more likely they are to demonstrate certain (psycho)linguistic behaviors (see “Issues with NATIVE SPEAKER when constructing comparison groups and conducting analyses” and Solís-Barroso and Stefanich (2019) for further discussion and examples of continuous vs. categorical language dominance measures). The interpretations offered by continuous analysis may also be easier to align conceptually with the knowledge that certain linguistic measures, like language dominance, are dynamic within the individual.

#### Individual Variation and Mixed-Effects Modeling

As discussed, there is a lot of heterogeneity in language experiences, and instead of attempting to make (artificially) homogenous groups, an alternative is to conduct individual differences analyses. Some approaches to language take such individual differences as the norm, even in relatively homogenous populations. For example, Dąbrowska (2013) argues from a usage-based perspective that what ends up looking like language-wide grammatical constraints are likely based in the cognitive biases of a subset of individuals, which then get amplified through patterns of transmission and use. Individual differences analyses often focus on factors such as working memory/attention, print exposure, or categorization gradience as predictors of linguistic behavior, predicting long-distance dependency resolution (e.g., Nicenboim et al., 2015), pronoun comprehension (Langlois and Arnold, 2020), or processing of phonetic cues (Ou et al., 2021), respectively. These types of analyses can also be applied to investigate the role of language exposure/use on linguistic knowledge/behavior.

Mixed-effects models, in which random effects allow for sub-group differences (as well as stimulus-derived variation), have become the norm in psycholinguistics. In particular, random by-participant intercepts and slopes account for variation at the individual level, which is typical in (psycho)linguistic data (see Barr et al., 2013). However, beyond simply “factoring out” individual-level variation that is not of interest to the research question, it is also often informative—and potentially crucial—to analyze individual response patterns in addition to group patterns. To illustrate, Tanner et al. (2013) found that while electrophysiological responses at the group level showed a biphasic pattern, no single individual showed that pattern but rather either an N400 or a P600 effect. In line with the individual differences approach, the authors argue that this demonstrates how new insight can be gained “when the cross-subject variability is treated as a source of evidence rather than a source of noise” (Tanner et al., 2013). One method to analyze individual variation is to use random effect coefficients output by mixed-effects model as the response variable, an approach that is increasingly common, for example, in individual-level correlation analyses of speech production and perception patterns (e.g., Pinget et al., 2020; Voeten, 2020). Overall, this approach has the advantage of moving away from categorical thinking and moving toward understanding underlying factors and mechanisms in language processing, which is abound with meaningful variability (e.g., see Yu and Zellou, 2019 for an individual differences approach to phonological processing).

#### Multivariate Data and Dimensionality Reduction

One way to handle large amounts of detailed demographic and language experience data collected from a questionnaire is to use dimensionality reduction techniques, like factor analysis or principal components analysis, to distill the data from many continuous measures into a smaller, more manageable number of relevant, orthogonal continuous factors. These factors can then be used as predictors in a regression model. Especially, when dealing with a sociolinguistic context which might be unfamiliar or understudied, allowing the relevant predictors to be inferred from the data in a principled manner, as opposed to data fishing, might be a desirable approach. This allows researchers to model language experience factors without defaulting to researcher-imposed categories; it could be that categories emerge from the data, but this allows that information to be inferred rather than imposed.

For example, Luk and Bialystok (2013) examined the responses of a highly heterogeneous group of 110 bilingual individuals (defined as individuals who had experience using two languages on a daily basis, with English being the dominant language of the community) to an English proficiency and self-report questionnaire, the Language and Social Background Questionnaire (LSBQ). Participants also completed the Peabody Picture Vocabulary Task-III (Dunn and Dunn, 1997) and the Expressive Vocabulary Task (Williams, 1997). Taking the many measures they collected, they conducted a factor analysis which found that language proficiency and bilingual usage are two continuous factors which should be included in models characterizing bilinguals (at least in a context where there is a dominant language in the society at large). For details of their analysis, we direct the reader to their paper, but this provides an example of how such analyses can be used to address theoretical questions about a heterogeneous group of participants. Other related analyses, such as principal components analysis, have long been used in studies of typology and variation (e.g., Abdi and Williams, 2010).

#### Emergent Groups and Clustering

An alternative approach to multivariate data, rather than identifying latent variables, is to use clustering techniques to classify individuals and identify emergent groups. According to Garcia-Dias et al. (2020), clustering is “a type of unsupervised learning which aims to find the most natural way of grouping a dataset” based on similarity across various dimensions. Clusters can then be interpreted by the researchers based on the contributing variables, and external validation can further be conducted to assess the extent to which each emergent cluster aligns with independently known variables. In other words, researchers can use clustering as a data-driven approach to identifying groups, if they exist, based on language experience or behavior rather than rely on predetermined and/or dichotomized categories based on ‘nativeness’ or other constructs.

Clustering can be used to identify “natural” groups (in a more informed manner than techniques such as median split) which are applied to test hypotheses or make comparisons. In some cases, groups can be predicted. For example, Novick et al. (2014) used clustering to identify “responders” and “non-responders” to an *n*-back task to investigate how responsiveness to cognitive control training influences recovery from misanalysis of sentence structure (i.e., garden-path sentences). In other cases, groups can be fully emergent. Chiarello et al. (2012), for instance, used clustering to identify four distinct subgroups of college-aged readers based in part on reading skill and then investigated the neurological correlates. Clustering is also generally useful for exploratory aspects of the research process, such as identifying individuals with similar response patterns to aid in data interpretation (e.g., sentence comprehension ability in aphasiac individuals or mono- and multilingual children, Caplan et al., 1985; Filippi et al., 2020). In addition, external validation can be a way of both exploring the data and testing hypotheses, often *via* the lens of individual differences (see “Individual Variation and Mixed-Effects Modeling”). In a study of second dialect acquisition, Voeten (2020) provides an example of using residential history as an external variable to examine the extent to which migrants who moved from Belgium to the Netherlands had adopted more Netherlandic-like vowels or retained more Flemish-like vowels (i.e., whether migrants’ vowel production measures clustered with those externally identified to be raised in the Netherlands or Flanders).

## Actionable Recommendations

Like Section “Assessment and Implications of Current Practices”, our actionable recommendations are divided into three sections that correspond to different stages of conducting research: conceptualization of research questions, characterization of language experience in experimental materials, and analysis of data. We begin with suggestions on how to conceptualize research questions and encourage a pause for reflection about the underlying assumptions of experimental constructs. The remainder of the section proposes ways to move beyond NATIVE SPEAKER with regard to various aspects of an experiment, including participant recruitment, stimuli development, task design, and assessment or screening question selection as well as during data processing and analysis. Throughout, we offer concrete actions to take along with illustrative examples to help readers apply these recommendations to their own research. However, not all laboratories or researchers have access to the same resources, whether that be physical space, time, funding, personnel, populations of interest, etc., and may not be able to follow all of these recommendations. We encourage researchers to do the best they can give their constraints. Researchers should prioritize the recommendations that best suit their research questions and experiments (see “Alternatives to NATIVE SPEAKER in Conceptualization” for help identifying which aspects of NATIVE SPEAKER are important for one’s research).

### Alternatives to NATIVE SPEAKER in Conceptualization

**Figure 1:**
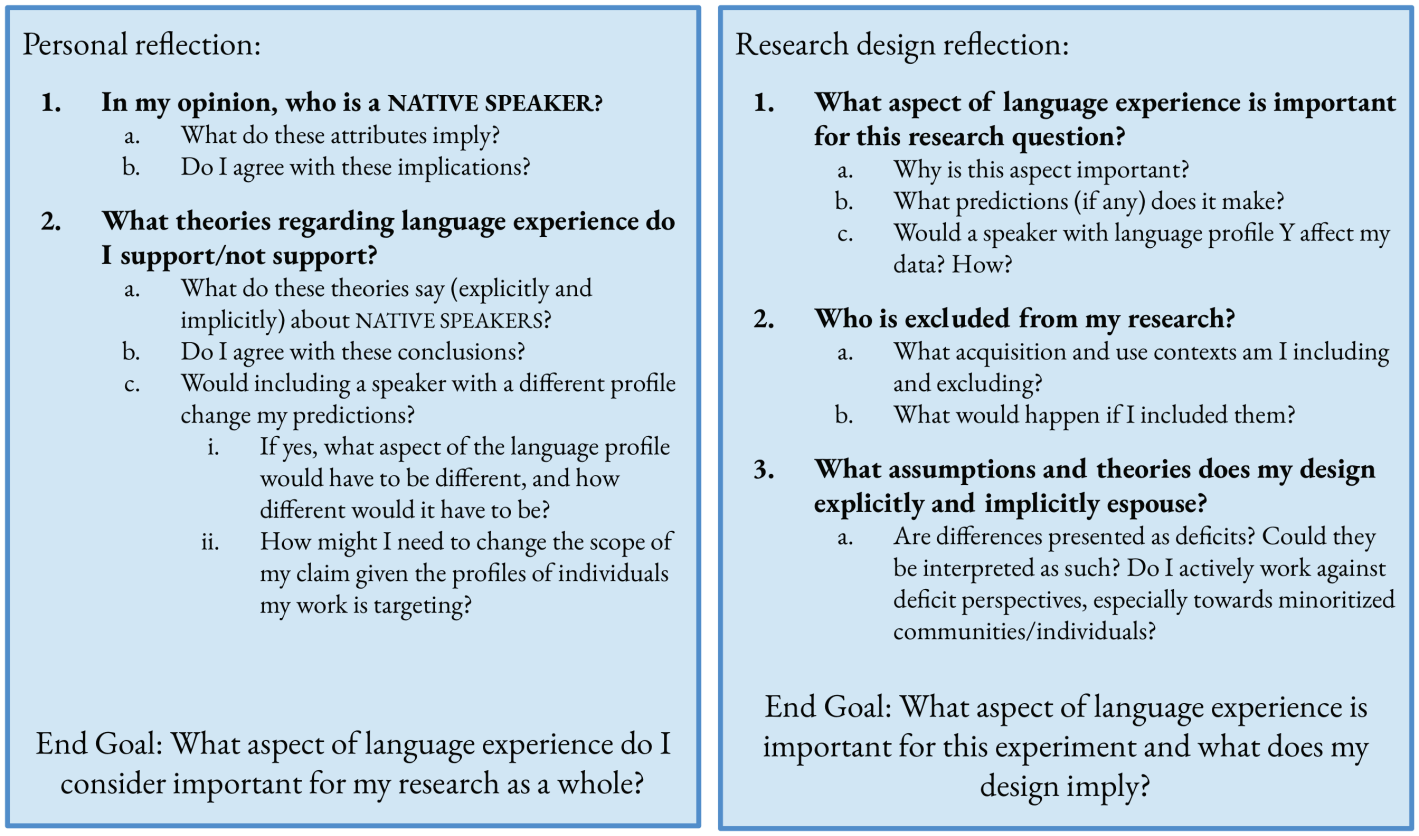
Reflection and conceptualization questions to ask during the research process. Please note that we are not encouraging researchers to define NATIVE SPEAKER in order to aid in continuing to use the term, but instead to think about the more granulated aspects of language experience that they use NATIVE SPEAKER to mean. This is an iterative process, and researchers will benefit from engaging in this process throughout the research process and their careers.

Before designing an experiment, researchers need to take time to think critically about both their own assumptions about NATIVE SPEAKERS and the questions they want to investigate. Understanding one’s own biases before engaging in research will result in more ethical and reproducible science. Throughout this questioning process, we encourage researchers to keep a log of the questions they ask and their answers. This log can help when writing up one’s theoretical viewpoint in a paper, explaining why or why not one included a variable in an experiment, as well as to see how one’s viewpoint has changed over time. See Figure 1 for a consolidated list of suggested questions.

Please note that we are not encouraging researchers to strictly define NATIVE SPEAKER and continue using the term, but instead to think about the more granulated aspects of language experience that they use NATIVE SPEAKER to mean. This is an iterative process, and researchers will benefit from engaging in this process throughout their career.

#### Reflection

First and foremost, we recommend that researchers ask themselves what their assumptions about NATIVE SPEAKERS are. This will help pinpoint the aspect(s) of language experience they are interested in and which they think are relevant for their overall research. To do this, researchers could start by concretely defining NATIVE SPEAKER in their own terms. For each of the attributes that the researcher lists, they should ask what the attribute implies. For example, if a researcher lists that a NATIVE SPEAKER “learned language X before age Y,” then this attribute implies a critical period. The researcher should evaluate whether or not they agree with this implication, determine if it is an important aspect of language experience, and update their definition accordingly.

Researchers should then evaluate the theoretical beliefs that they hold about NATIVE SPEAKERS. One should ask themselves what theories they do or do not support and list the assumptions that those theories make about language experience. Then, the researcher can determine if they agree with those assumptions. The researcher may notice a trend among the assumptions that they agree with. For example, a researcher may support theories that imply that someone is a NATIVE SPEAKER of X if X was the language of instruction at school. This indicates that the researcher views language of instruction as an important aspect of language experience. Crucially, a researcher should then ask “Would including a participant with a different language profile change my theoretical predictions?” If the answer is “Yes,” then the researcher should consider what aspect of a participant’s language profile would have to be different (and how different) for the researcher to see a change in their predictions. Once a researcher has completed this line of questioning, they will be more equipped to determine the aspects of language experience they consider to be important for their research as a whole. Identifying one’s underlying assumptions can then inform recruitment, experiment design, and data analysis.

During this reflection, some may feel resistance to shifting away from NATIVE SPEAKER, which may have been a functional or central concept in their past research. We do not deny that certain commonalities may be observed among those broadly considered NATIVE SPEAKERS (or L1 speakers) by researchers. However, these communalities may be more precisely captured by factors of language history, proficiency, and/or identity, meaning that a move away from fuzzy categories is a move toward clarity of mechanisms. Given this context, we ask that researchers consider carefully whether the implicit and explicit exclusion of marginalized and minoritized populations through the usage of this term is necessary or justified for their particular research program or questions. It is crucial for researchers to be explicit about the types of language experience which are important to their research questions in order to avoid reproducing normative assumptions about who gets to be a NATIVE SPEAKER. By pulling away from the term NATIVE SPEAKER as a proxy variable, researchers can reduce harm and begin to better align their research and theory with non-normative contexts of language learning and use.

#### Research Design

After researchers have analyzed their own beliefs, they can begin designing experiments that take into account their assumptions. Researchers often begin the research conceptualization process with a general question that they translate into a specific, operationalized hypothesis. If researchers have gone through the initial reflection process, then they already know what aspects of language experience are relevant for their research, and what aspects are important for specific questions. However, it is important to ask follow-up questions for each new experiment. Researchers should engage in this process for each new experiment that they design.

The first question a researcher should ask is as follows: What aspect of language experience is important for this specific research question? If they are not able to identify an aspect, they should go through the self-exploration process in Section “Reflection” again. Once researchers have identified what aspect of language experience is important for their question, they should identify why it is important. This question has two goals: (1) It situates the work in a theoretical context, and (2) it ensures that the construct is relevant. When researchers tie their experiments to broader theories, they should again evaluate what these theories imply about NATIVE SPEAKERS and whether or not they agree. If researchers are not able to identify why a construct is important, then they should consider using a different aspect of language experience (though note that this may not be relevant for exploratory research, where there may not be strong evidence for the importance of a construct). At this stage, it is also beneficial to determine what predictions come from a researcher’s chosen aspect of language experience. While this should be a standard part of experiment design, thinking through possible results is particularly beneficial in the case of research derived from the concept of NATIVE SPEAKER because it can indicate whether existing constructs are informative and relevant.

One should also consider whether including speakers with different language profiles would affect the data, and if so, how. Similar to whether a different language profile would impact theoretical predictions, this question evaluates both the scope of the empirical question and how susceptible the experiment design is to heterogeneity within speaker groups. If slight variations in speaker profile change the predictions, then the natural heterogeneity of participant groups will lead to differences in results. Exploring how one’s data would change by including participants with different language profiles may reveal relevant aspects of language experience the researcher had not previously considered.

Considering how language profiles affect data leads nicely to the next important question: Who is the researcher *excluding* and why? If researchers are excluding a group due to a specific aspect of language, we encourage researchers to tailor their screening questions to *measure* that aspect of language instead of blanket-eliminating a widely heterogeneous group of subjects (see “Alternatives to NATIVE SPEAKER in data processing and analysis” for more information). Researchers should consider what their data and predictions would look like if they did include these speaker groups and contexts.

Lastly, researchers should dedicate a considerable amount of time to evaluate how they structure their research questions and situate them in a theoretical framework. As discussed previously, many existing theories make assumptions about NATIVE SPEAKER and inherently imply other debated frameworks. By carefully examining the assumptions underlying theory, researchers can avoid vagueness and enhance replicability. Assumptions about NATIVE SPEAKER as a concept may also exclude certain participants from research, perpetuating harmful stereotypes and deficit models. We call on researchers to establish their research in explicit theories of language learning, exposure, and usage. Within this solid framework, researchers can then clearly state what they are testing and how it relates to the variables within the theoretical framework. The participants’ explicit, specific, and measurable language experience should be the driving variables in theory development and experiment design.

### Alternatives to NATIVE SPEAKER in Recruitment, Tasks, and Surveys

Various aspects of an experiment may use the term NATIVE SPEAKER or assume the construct as given. In this section, we provide best practices to move beyond this when designing materials for participant recruitment, experimental tasks, and surveys, as well as when reporting methodological details in a publication.

#### Participants and Groups

First and foremost, we recommend that researchers actively avoid describing participants as ‘(non-)native speakers’ in any part of the experiment, including the use of this concept as a criterion for assigning participants to target and comparison groups. Instead, the best practice is for researchers to, based on their specific research question, specify targeted aspects of linguistic experience in detail (for recommended reflection questions, see “Alternatives to NATIVE SPEAKER in Conceptualization”). These concrete characteristics can then be used in recruitment and analysis, promoting clarity and transparency.

For example, when asking about the effect of language experience on acceptability of resumptive pronouns in Egyptian Arabic, one might construct comparison groups differently based on one’s particular assumptions about the role of language experience. If a researcher thinks that speaking Egyptian Arabic as the primary language is what matters, that would be one way of recruiting and characterizing a comparison group. Alternatively, if a researcher thinks age of acquisition is what matters, recruitment could take place based on whether participants had grown up speaking Egyptian Arabic. Crucially, these different hypotheses would result in potentially different groups of participants, and asking about ‘native speaker’ status would result in a third, less-specified group.

Some of this information can be collected after the experiment is completed, in a post-experiment questionnaire, but, when possible, conducting norming studies (finding out what types of language experience are common in the local context), looking to census data, or making hypotheses based on ethnographic or sociolinguistic research is also a good practice (cf., Chung et al., 2012; for recommended questions to consider, see “Surveys and Recruitment”).

#### Stimuli and Norming

Likewise, researchers should make sure to stipulate beforehand, and collect detailed information about, the demographic and language experience characteristics of individuals recruited to record auditory stimuli or to participate in norming/coding of experimental items. The experimenters can then use this information in the interpretation and discussion of results. Ensuring that this information is also reported in publications serves to transparently communicate the context for readers to potentially replicate the study materials.

Crucially, we are not asking that researchers necessarily report any and all information they might have (though reporting such information in supplementary materials or appendices is an option); rather, we ask that researchers aim to have clear, *a priori* expectations about which aspects of language experience are relevant for their particular research context (see “Surveys and Recruitment” for some ideas). In the case of recording auditory stimuli, some relevant elements to report are (i) whether the person is from the same community as the participants, (ii) how their language experience might match or deviate from that of the participants, and (iii) when possible, any information about whether and how that might be perceived by the participants.

For example, if a researcher is conducting a study on constituent ordering preferences in Hindi-Urdu speakers living in the United States, rather than reporting that ‘a native speaker of Hindi-Urdu was recorded for auditory stimuli,’ a better practice would be to report details, including the location and linguistic context in which the speaker grew up and currently resides, the speaker’s regional/cultural variety of Hindi-Urdu, and their language usage practices, including potential multilingualism. Similarly, for a researcher conducting a study on processing of ‘foreign-accented’ speech, rather than reporting that ‘ten non-native speakers of English recorded sentences,’ it would be better practice to report details (in the main text or in a table) about each speaker’s regional background, linguistic history, and perceived degree of accentedness.

#### Tasks and Assessments

When choosing assessments to characterize participants, researchers should keep the following question in mind: Is this assessment measuring ‘nativeness’ as I operationalize the term, or is this assessment capturing others’ ideologies about what a ‘native speaker’ is? When we ask participants to self-report their “native” status, in reality, the results capture their ideologies of what a ‘native speaker’ is and whether they fit into that definition. The same reflection question must be used when considering apparently objective measures. Take, for example, ‘nativeness’ accent ratings. In this type of assessment, speech samples from the participants are given to raters (usually members of the target speech community) who then judge how “native” a speaker sounds. While accent ratings appear to be collecting objective data, in reality, this measure too is capturing the rater’s subjective ideologies about what a ‘native speaker’ should sound like and whether their perception of the speaker matches their ideologies of nativeness.’

To avoid assessing ideologies about NATIVENESS and leaving room for interpretation of what this term means, we recommend that our assumptions as researchers about NATIVENESS be made explicit in the assessments we select. If, for example, a researcher were to be interested in determining whether participants grew up speaking the “native” language at home and at school during early childhood, rather than asking an ambiguous question, such as “Are you a native speaker of German?” one could ask more targeted and explicit questions, such as “Did you grow up only speaking German and spoke it at home and school?” (see Table 2 for more examples). The same reformulation can be made to assessments like the ‘native accent’ ratings. For example, instead of asking “Does this person sound like a native speaker of Spanish?” a more specific question could be “Does this person sound like they grew up in Mexico?”, “Does this person sound like a local?”, or “Does this person sound like they are a monolingual speaker of Spanish?”. The same caveats about limiting participant pools apply here; we are not advocating that researchers stick to ostensibly monolingual participants. Rather, this is a demonstration of less harmful and more accurate ways of asking questions about language experience.

As for assessments of proficiency, we have given examples throughout this paper of how uncritical use of proficiency measures can be damaging and misleading. However, if a notion of proficiency is crucial to the research question, we advise that researchers keep in mind that proficiency can vary across domains (e.g., speaking, understanding, reading, and writing), and we urge that proficiency be properly contextualized, taking language access and structures of oppression into account. Much like Ortega (2020) suggests in her social justice-focused review of heritage language development, psycholinguists can guard against assumptions of proficiency that privilege hegemonic monolingual norms by ensuring their research considers the myriad ways proficiency develops and is demonstrated across a person’s life course. Considering how local language ideologies and institutional opportunities and impediments influence participants’ language use in different domains will lead to a more nuanced picture of proficiency.

#### Surveys and Recruitment

The questions in Table 2 give examples of how researchers might question and characterize aspects of language experience relevant to their research purpose without relying on implicit assumptions about ‘native speakerhood.’ Consider that while focusing on a small number of categorical distinctions may be useful for recruiting participants in a straightforward and practical way, it may be more helpful for theorization and analysis to capture these variables with more gradient measures, as discussed in Section “Complicating NATIVENESS in Data Processing and Analysis”.

Whether or not one’s study design involves explicit predictions or analyses based on participants’ language experience, the sociolinguistic context should inform how researchers choose questions for recruiting and characterizing participants and how they interpret and report responses. Norms of multilingualism and schooling in the local context may affect to what degree a given question about language experience will generate a homogeneous sample. The desirability of homogeneity will depend on the purposes of the study, but be cautious of the trade-off between the homogeneity of a given sample and the generalizability of an observed effect. We caution against making broad claims about language organization and behavior based on studies drawing on samples from relatively homogenous (and WEIRD) speech communities. However, precisely, reporting the language and cultural background of one’s participants (as discussed in “Participants and Groups” and “Stimuli and Norming”) can help inform directions for expansion in the future research.

Here, we give some suggestions for the types of information which might be relevant to take into account when contextualizing results, and therefore the types of information which researchers can ask about and report in publications. For example, is the language or variety stigmatized, either locally or by the larger society? Is the community minoritized (even if the language/variety is not stigmatized)? Are the speakers (and perhaps by extension particular linguistic features) racialized? What is the incidence of multilingualism in the community? How does schooling look typically, and what are the associated language policies? What counts as being multilingual for the local context? Are the language boundaries which linguists can perceive relevant for the speakers themselves (cf. Otheguy et al., 2015)? Looking to the boxes in this paper can give some examples of how speakers and communities might vary, and the types of information which would be relevant for understanding how speakers may or may not be typical of the populations of interest.

### Alternatives to NATIVE SPEAKER in Data Processing and Analysis

Rather than comparing groups of participants based on NATIVE SPEAKER status, we recommend that researchers compare response measures based on more transparent and targeted variables of language experience relevant to their research question. As part of this move away from categorical analyses, we encourage researchers to take an individual differences perspective to foreground individual-level patterns in their data. There are several ways this could be implemented. In a context where researchers recruit from the same pool of participants, expecting heterogeneity of experience but not expecting qualitatively separable groups, one could examine data for apparent outliers and attempt to interpret these based on language experience factors. Alternatively, one could examine whether the dependent variables are multimodally distributed, and conduct *post-hoc* analyses connecting those patterns with information about language experience. Finally, even in contexts where there might be qualitatively separable groups, researchers can examine how relevant language experience factors might contribute to how individuals do or do not map on to group-level patterns.

We briefly direct the reader to methods of analysis which align with the theoretical moves we are advocating to accurately and fairly represent our research populations, namely, using continuous variables instead of categories in linear regression and taking into account individual differences using mixed-effects modeling (Regression Analysis), as well as using dimensionality reduction techniques on many continuous variables and using clustering techniques to split the data into observed rather than predetermined groups (Multivariate Data). We recognize that these statistical tools may not be available or applicable to all researchers, particularly those who work with smaller sample sizes (e.g., due to constraints of the participant population); in such cases, it might be necessary to consider whether non-parametric statistical methods or qualitative analyses are more appropriate.^9^ Our recommendation is that researchers thoughtfully consider using the alternative methods and approaches that are relevant for their particular research context, given their practical limitations.

#### Regression Analysis

Regression analysis is widely used in psycholinguistics, and it allows us to account for continuous predictors. To take a concrete example, let us say Dr. A is interested in differences in lexical processing based on language experience (determined through the researcher’s own reflection during conceptualization; see “Alternatives to NATIVE SPEAKER in Conceptualization”). Dr. A may recruit participants who are all currently residing in Germany and have either German or English as their L1. Along with a German lexical decision task, they ask their participants about their age of acquisition and self-rated language usage and proficiency in a post-experimental questionnaire. One or more of these variables can be added as continuous predictors into a regression model.

Familiarity with regression-based approaches to analysis is necessary when dealing with continuous predictors, and unfamiliarity and lack of training with regression-based statistics may explain some of the continued “categorical thinking” in the field. We recommend that researchers follow the current norm for modeling psycholinguistic data, which is to use mixed-effects regression models (also called random-effects, hierarchical, and multilevel models; Gelman and Hill, 2006, p. 2) to control for participant heterogeneity (i.e., individual variability). To continue the example above, Dr. A should, at the very least, include by-participant random intercepts, which allow individual participants to be modeled with different mean response times, as well as by-participant random slopes, which allow for individually variable patterns of response time to experimental conditions (e.g., real vs. nonce words). Some resources for learning and using regression analyses include Winter (2019) *Statistics for Linguists: An Introduction Using R*, Baayen (2008) *Analyzing Linguistic Data: A Practical Introduction to Statistics using R*, and Gelman and Hill (2006) *Data Analysis Using Regression and Multilevel/Hierarchical Models* (see also Barr et al., 2013 and Bates et al., 2018 on conventions for constructing mixed-effects models).

#### Multivariate Data

Dimensionality reduction and clustering techniques allow one to identify patterns between several variables simultaneously, which can be particularly helpful when handling and making sense of large amounts of language experience data. When multiple predictors are considered in regression modeling, correlations between predictors (i.e., collinearity) can cause adverse consequences for analysis. This is something to keep in mind when dealing with many language experience variables, because we may expect several variables of language experience to correlate with each other, in addition to being correlated with the response measure. For example, age of acquisition is often correlated with measures of proficiency (Birdsong, 2005). So, Dr. A may choose to select only one of the highly correlated variables in their data set to enter as a predictor in the model (especially when these variables and their consequences are not of primary interest), or they may choose to perform more sophisticated multivariate statistical techniques to work around this problem. Tomaschek et al. (2018) address several strategies for diagnosing and addressing collinearity in multivariate linguistic data, of which dimensionality reduction is one solution.

Dimensionality reduction techniques include factor analysis (FA) and principal components analysis (PCA). These techniques are similar, but different in their approaches and assumptions. PCA is usually preferred when the goal is simply to reduce correlated observed variables to a smaller set of composite variables, whereas FA is preferred when the goal is to detect underlying factors influencing the responses on the observed variables (for a more detailed but still approachable comparison of these methods, see Brown, 2009). Luk and Bialystok (2013) provide their reasoning for using FA instead of PCA in precisely these terms; they were interested in an assumed underlying causal relationship between the observed variables and the latent factors of interest, which were related to aspects of bilingual experience. Hypothetically, Dr. A may choose to do a factor analysis and find two latent factors which they interpret as roughly corresponding to language history (age of acquisition, proficiency, and past usage) and current language usage. For an introduction to factor analysis, see Thompson (2004) and Formann (2014) for latent class analysis. For an introduction to FA and PCA (among other multivariate analysis techniques) in R, see Chapter 5 of Baayen (2008).

Whereas dimensionality reduction aims to identify and reduce irrelevant or redundant variables, clustering aims to identify natural groupings of data points based on similarity. Language experience variables (e.g., from a screening task or language experience questionnaire responses) may not always be evenly distributed across a continuous range. Data may instead be multimodal such that certain participants are particularly similar to each other, representing groups who are qualitatively different, at least in that particular context or participant sample. In this case, participant groups could be identified in a data-driven way *via* cluster analysis, interpreted relative to the language experience profile per cluster, and then used as a categorical variable in planned analyses. To illustrate, if Dr. A performed a cluster analysis on each participant based on their reported German age of acquisition, usage rating and proficiency rating, this may hypothetically result in three clusters with distinct language experience profiles: early learners with high usage and high proficiency, later learners with high usage and high proficiency, and later learners with low usage but high proficiency.

Before clustering, diagnostics should be used to confirm that the data indeed are multimodal. There are various types of cluster methods, including the broad classes of hierarchical methods, where each individual begins as its own cluster and is grouped with progressively more clusters (e.g., Ward’s method) and partitioning methods, where number of clusters are prespecified and individuals are assigned to a particular cluster (e.g., K-means). For more information about cluster analysis, Jain (2010) provides an overview of the method (see also Chapter 5 of Baayen, 2008). Other resources include Garcia-Dias et al. (2020), which focuses on K-means clustering, and Clatworthy et al. (2005), which reports on how clustering analyses have been used in health psychology.

## Conclusion

It is clear that the concept of NATIVE SPEAKER can be harmful to both psycholinguistic research and the populations we study. The term NATIVE SPEAKER has been problematized in different fields, and some solutions have been to repurpose the term. However, these approaches simply redefine NATIVE SPEAKER to include or exclude certain populations (Costello et al., 2008; Benmamoun et al., 2013; Rothman and Treffers-Daller, 2014) and many of the same problems remain. We encourage researchers to abandon the term altogether and join our colleagues in adjacent fields to adopt a more nuanced view of language experience. We anticipate that the recommendations in Section “Actionable Recommendations” can improve research at all stages: theory construction, experiment design, participant recruitment, stimuli creation, and data analysis. To reiterate, researchers should explicitly define which aspects of language experience they are investigating, recruit individuals and select assessments that are targeted to these aspects, ask specific questions to understand participants’ language experience, account for the sociolinguistic context in which the questions are asked and how they are interpreted, and use gradient measures instead of categorical variables when relevant.

As we reflect on these recommendations, recall the point made in Section “Actionable Recommendations”—as researchers may not be able to practically implement all of these all at once, they should prioritize the changes which are most relevant for their research questions and the contexts in which they work. However, individuals and individual laboratories cannot on their own be responsible for shifting away from this concept and the associated baggage. We recognize there are structural barriers to implementing some of these recommendations, such as expectations of reviewers, access to funding to collect requisite data, and necessarily small sample sizes when working with certain populations. Of course, we psycholinguists are part of these structures to different degrees and can work within our spheres of influence to make a difference. This could look like advocating for less harmful ways of characterizing and recruiting participants as part of local/institutional ethics boards, program committees, journal editorial boards, and in classroom or other training contexts. Structural changes are necessary and must accompany the individual-level changes which are the focus of this paper. We hope these recommendations will not only improve research design and analysis, but also contribute to the co-creation of a more just and inclusive field.

## Author Contributions

All authors contributed to the conceptualization and writing of this paper. The most substantive contributions of each individual are listed as follows. LC: Vagueness, Issues With NATIVE SPEAKER at the Stages of Conceptualization and Design, Issues With NATIVE SPEAKER When Constructing Comparison Groups and Conducting Analyses, Complicating NATIVENESS in Conceptualization, Continuous Variables and Linear Regression, Multivariate Data and Dimensionality Reduction, Emergent Groups and Clustering, Participants and Groups, Stimuli and Norming, Tasks, and Assessments, Regression Analysis, and Multivariate Data. DB: Issues With NATIVE SPEAKER When Constructing Comparison Groups and Conducting Analyses, Continuous Variables and Linear Regression, Individual Variation and Mixed-Effects Modeling, Multivariate Data and Dimensionality Reduction, Emergent Groups and Clustering, Surveys and Recruitment, preparation of Table 2, Regression Analysis, and Multivariate Data. NV: Continuous Variables and Linear Regression, Complicating NATIVENESS in Conceptualization, Reflection, Research Design, preparation of Figure 1, and Conclusion. CS-B: Vagueness, preparation of Table 1, Complicating NATIVENESS in Recruitment, Tasks, and Surveys, and Tasks and Assessments. AM: Harm, Issues With NATIVE SPEAKER at the Stages of Conceptualization and Design, and Complicating NATIVENESS in Recruitment, Tasks, and Surveys. SN: Harm, Issues With NATIVE SPEAKER at the Stages of Conceptualization and Design, Issues With NATIVE SPEAKER When Constructing Comparison Groups and Conducting Analyses, Complicating NATIVENESS in Conceptualization, Complicating NATIVENESS in Recruitment, Tasks, and Surveys, Multivariate Data and Dimensionality Reduction, Participants and Groups, Stimuli and Norming, Stimuli and Norming, and Conclusion.

## Conflict of Interest

The authors declare that the research was conducted in the absence of any commercial or financial relationships that could be construed as a potential conflict of interest.

## Publisher’s Note

All claims expressed in this article are solely those of the authors and do not necessarily represent those of their affiliated organizations, or those of the publisher, the editors and the reviewers. Any product that may be evaluated in this article, or claim that may be made by its manufacturer, is not guaranteed or endorsed by the publisher.

## Footnotes

^1^Throughout this article, we use NATIVE SPEAKER in small caps to denote the construct. We use ‘native speaker’ in single quotes to refer to hypothetical uses (as in, the so-called native speaker). We reserve “native speaker” in double quotes for direct quotations. In all cases, we set the term NATIVE SPEAKER apart in order to signal that it is not used in a manner that assumes it as the default, unmarked state.

^2^Language *experience* is conceptualized broadly, encompassing all aspects of linguistic identity, proficiency, usage, input, output, language contact, etc. In comparison, we use language *history* to refer specifically to aspects of language development which are included in more “traditional” accounts of language acquisition, such as age, order, or context of acquisition. Crucially, we consider these factors to be subsumed under language experience.

^3^As the distinction between language and language variety is socially constructed, here and elsewhere, what we say about monolingualism and multilingualism could also apply to monolectalism (using one variety of a named language) and multilectalism (using multiple varieties of the same named language).

^4^See discussion in Fisher et al. (2018) of how researchers’ priorities in selecting Deaf participants who fit a particular profile may have influenced ideologies of belonging in certain (American) Deaf communities.

^5^Here, we find it relevant to highlight another way that the term NATIVE is commonly used, that is, to refer to the First Nations of North America. This adds another facet of ambiguity in how this term might be used and interpreted.

^6^For the purposes of this paper, we are construing psycholinguistics broadly, to include those who research sentence processing, speech perception, language development, and any other research area in which experimental methods might be used to investigate language perception, production, learning, and/or comprehension.

^7^n.b., We are not intending to call out any particular authors with these examples—they are illustrative of the norms in psycholinguistics as we see them.

^8^See also Rochat (2010) who argues that psychologists need to go beyond simply diversifying participant populations and points out that WEIRD is itself a binary category which does not capture all the facets of overrepresentation. In addition, Clancy and Davis (2019) point out that WEIRD is racialized, in that it almost always means white, which adds another important dimension to which types of participants are systematically included and excluded.

^9^Due to disciplinary norms and training, psycholinguists may not be used to thinking about qualitative analyses, including descriptive statistics or visual interpretation (as relevant), as an option. We are not suggesting that qualitative analyses are inferior or a “last resort,” but rather they yield different types of information. We hope psycholinguists can consider this an additional tool in their analytic toolkit. As discussed in the conclusion, we recognize this would require structural changes beyond the control of an individual, but being able to include smaller populations without obscuring meaningful variation would be a great advantage.
